# Miscellanies

**Published:** 1870-02

**Authors:** H. Scott

**Affiliations:** Lancaster, O.


### MISCELLANIES.

BY H. SCOTT.

*Manipulation.*—It will not be expected that all manipulators of the mouth can be equal in tact and ability; in fact, there will be found the same differences in skill and execution among Dentists that exist in the mechanic arts; in music, poetry, or anything else that man attempts to do. All are not gifted alike. Some can no more than imitate what others do well with little effort. In this there will be no difference of opinion. The ability to plan, and the ability to execute, are separate talents. Many minds are fruitful in origination, whose hands can do little in putting their designs into shape and artistic finish. This, also, will be conceded. But it is to be expected that men can, or will, find what they are best fitted for, so as to start out on the right road before entering upon life's active duties. This achievement is not likely to be reached.

But it is no part of our present purpose to analyze all men and find what they are fit for, and then assign to each his task. It is not our business to say that many have chosen Dentistry for their avocation who would have made better merchants, or farmers, or lawyers; but this much it is right to say: Those who by nature have been given abilities to become good mechanics, would, with equal effort, become good Dentists, generally, if due modifications be made for timidity. But there are many engaged in Dental manipulations, and, without attempting to sort them out, we will say some things that may help the good as well as the indifferent operator, if acted upon.

There is less in a greater array of instruments than there is in cool, calm and patient determination to succeed; and first of all is coolness. If a man can not so command his nerves and his philosophy, as to be unmoved by the petty annoyances that arise in the progress of filling a tooth, or adjusting an artificial denture, or even extracting a tooth, he will not likely please either his patient or himself. And we shall hold that a Dentist is to be wholly indifferent to the caprices, or fears, or suggestions of his patients. If he can not so feel, and so act, he will often fail. The great point is to comprehend what is to be done, and then feel that you can do it. And then again, Dentists manacle themselves by attempting too much machinery, and by trying to adopt this or that new suggestion, thus giving up their acquired advantages for some new way of doing a thing, only to find themselves embarrassed and defeated in attempting to do what they had often done before, successfully. Thus, through fear that moisture will get into their cavity—before they can finish a filling—they spend all their attention in trying to keep the water away, and blunder along amid harrassing circumstances, and at last make a bad filling. In an old standard author, the writer said: "*He is a bungler who can not remove the tartar from the teeth without making the gums bleed.*" This is nonsense to a practical man. What is required is to clear the calculus away; and the more the gums bleed the better. It is simply not necessary to lacerate the soft parts in an awkward use of instruments; and a man will be the best tooth extractor who knows the anatomy of the parts, the strength of his tooth and the adaptation of his instrument, and then deliberately, and with perfect self-reliance, does his work, forgetting for the time being the prescribed rules laid down in the books. We must be entirely self-reliant. Of course, written directions are not to be ignored; but they are not to displace our experiences

But not to be in a hurry, or impatient of time, or afraid of one's own ability; these are some of the secrets of success. And the examples of seeing how other people do things in their specialties, are aids to Dentists.

Thirty years practice has given me confidence in myself, but the way my workmen handled their tools while erecting my new residence, during the past year, gave me some lessons that have helped me to work easier and better. Simplicity in the number and variety of instruments is better than too much complication. To reach success in any of the manipulations about the mouth, the concentrated attention of the manipulator must go with the point of his instrument; and his eye must be there too, whenever it can peer to the spot. That which can not be done is not to be attempted; and to try to do a thing like somebody else says they do it, is to abandon a way of your own that has been successful, and given you, perhaps, conscious satisfaction. This will be wrong, generally, and the cause of failure. On the other hand, we are not to discard new suggestions because they are new; they are to be tested, to see what they are worth to us. Only in this way do we advance. But if one insists that the ten ounce lead mallet is the best thing to consolidate gold with, and you are doing good work with the two ounce wooden one, don't be too ready to make the change; for if you do change, you may find yourself away from home. It is not difficult to see that equally good fillings can be made with either, in different hands, or with the automaton, or by hand.

*Amalgams.*—I have not found much difference in the fitness of the various amalgams for tooth-stoppings, as they are now improved and sold. I submit a few lines on my manner of manipulating them. But first, I do not believe that good amalgam work is secured by manipulating in the palm of the hand; I am sure it is not. My method is to triturate in a mortar till there is perfect amalgamation, and then to take the mass through several washings—not less than three; and this I do by continual rubbing with the pestle. I remove the mass from the mortar after each washing, and wipe the mortar and pestle perfectly dry each time. When the fluid is no longer visibly colored, then the process is finished. I have found that a large amount of heavy rubbing, with the pestle, is required to make the solidest fillings; and this I am sure is one of the conditions of good amalgam work. I squeeze all the mercury from the metal that I can, through buck or chamois skin, softly dressed. In packing, I use heavy force, placing the metal in small pieces, and, as the surface softens under the packers, I scrape away the excess of mercury, and commence again with fresh pieces of the mass. I never can fill more than two, sometimes only one cavity with the same mixing, on account of the preparation becoming too dry to work. I have been astonished at the firmness of my amalgam fillings, and the fineness of the finish they take, both of which I attribute to the protracted trituration, the thorough washing, and the dry state in which

I use the metal. Fillings of amalgam, conducted in this way, do not contract perceptibly from the borders of the cavities. I do the work as well under the saliva as in the dry cavity. The wet makes no shade of difference in the goodness of the work. Try it, and you will see that I am right.

*Tin Foil.*—I continue to use, chemically, pure tin foil for cheap fillings; but I use it only in medium and small cavities on the lateral and crown surfaces of the bicuspids and molars, where oxydation is the least likely to take place. My method of using it is as follows: I prepare it in cylinders, or balls, as firmly manipulated out of the mouth as is compatible with driving it solidly to the walls. In packing, I use heavy malleting force, both with automaton and hand mallets—being always specially careful that the foil does not get out or mangled in the process. I make the metal as solid from the bottom to the surface as it is possible to do, and then chisel and file away to the required shape, and finish as fine as tin will take. I have found that tin fillings, when finely polished, resist oxydation much more than when left in an unfinished state; and I have been surprised, in some instances, at the amount of use they have endured without loss of substance. I think it more important to keep dry while filling with tin than with amalgam.

*Perplexities.*—How far may a Dentist compromise himself in acceding to the ignorance and caprices of occasional patients?

A lad called to have a permanent molor extracted, and, with peremptory injunction from his mother that the gum must not be cut. I sent him home. In an hour he returned, accompanied by his maternal parent. She had known "*a woman who had the gum cut, and bled to death.*" I assured her that such an occurrence could not be one in one million, and that no one could bleed to death now from tooth extraction, with the means at our control for arresting the bleeding. But she was inaccessible. She wanted the tooth out, but "*the gums must not be cut.*" I dismissed them. Did I do right? I could have taken the molar out without the lance; but is it right to let one's self down to such a whim?

A young miss came with a written note from her mother, requesting me to fill her teeth, but I "*must not file them.*" I requested the miss to ask her mother to call at my office; which she did on the following day. I explained that some of the front teeth would require the file. She was firm in her objection. It would "*break the enamel, and cause the teeth to decay faster.*" I suggested that the crumbling edges must be removed to guarantee good work; and asked her if she thought it could make any difference whether they were taken away with the file or the cutting instruments. I might "*cut them, but must not use the file.*" My patience was waning, and, as there was another case waiting in the sitting-room, I asked the daughter to please give up the chair. I don't know whether she found a man that she could order or not. The girl had my sympathies; the mother, pity.

A man of some ability, financially, sent his daughter to have an incisor filled. I found the four six year molars with pretty little cavities in the grinding surfaces. I suggested that they ought to be filled. She "*guessed father wanted her to have all done that needed doing.*" I filled them, and gave her the bill, which she asked for. The father came in a couple of days, and was terribly angry. I had "*bored holes in teeth that didn't need it, just to get a job.*" I receipted his bill, and handed it to him. But, oh no! he would "*pay what was right;*" he would "*pay for the front tooth.*" I made no reply, but turned to my work, and he went away. The mother came afterwards to have a compromise, by paying half the bill; "*perhaps the teeth did need plugging—some of them.*" I made no concessions, and got no money in the case, and never will.

*A Case.*—Miss L. had an exposed pulp. It was the first left superior molar. The cavity was from the front approximal surface. The tooth was firm, and in all respects, except the decay, in good condition. Miss L. was eighteen, and of sanguine bilious temperament, enjoying very good health. I prepared the cavity well, and filled very successfully with os-artificial. There was just the usual amount of twinging after application, and the tooth did good service for about eighteen months. Upon removing the zinc filling, at the end of that time, there was seen a solid bony arch thrown over the aperture, a little elevated in the center, and very hard. The color of the crown was natural, and indicated a living tooth, but there was found no sensibility of the dentine. I filled the cavity solidly with tin foil, using mallet force. It was a success, and would be under similar circumstances. My next card in the papers, said: "*Teeth with exposed nerves saved alive.*" For two years more nothing was wrong; but at last she came, with a frightfully swelled face, which had tortured her for thirty hours. The tooth was removed and split open, and every indication was present that the nerve had been dead a long time—perhaps ever since the oxychloride was applied. I think so now. It was her catamenial period.

LANCASTER, O.

